# Quantitation of DNA methylation in Epstein-Barr virus–associated nasopharyngeal carcinoma by bisulfite amplicon sequencing

**DOI:** 10.1186/s12885-017-3482-3

**Published:** 2017-07-17

**Authors:** Weilin Zhao, Yingxi Mo, Shumin Wang, Kaoru Midorikawa, Ning Ma, Yusuke Hiraku, Shinji Oikawa, Guangwu Huang, Zhe Zhang, Mariko Murata, Kazuhiko Takeuchi

**Affiliations:** 1 Department of Environmental and Molecular Medicine, Mie University Graduate School of Medicine, 2-174, Edobashi, Tsu, Mie 514-8507 Japan; 2 Department of Otorhinolaryngology, Head and Neck Surgery, Mie University Graduate School of Medicine, Tsu, Mie Japan; 3 Department of Otolaryngology Head and Neck Surgery, First Affiliated Hospital of Guangxi Medical University, Nanning, China; 4 Graduate School of Health Science, Suzuka University of Medical Science, Suzuka, Mie Japan; 5 Present address: Department of Research, Affiliated Tumor Hospital of Guangxi Medical University, Nanning, Guangxi China; 6 Present address: Center for Oral Biology, University of Rochester Medical Center, Rochester, NY USA

**Keywords:** DNA methylation, Methyl-capture sequencing, Bisulfite amplicon sequencing, Nasopharyngeal carcinoma, Epigenetic mark

## Abstract

**Background:**

Epigenetic changes, including DNA methylation, disrupt normal cell function, thus contributing to multiple steps of carcinogenesis. Nasopharyngeal carcinoma (NPC) is endemic in southern China and is highly associated with Epstein-Barr virus (EBV) infection. Significant changes of the host cell methylome are observed in EBV-associated NPC with cancer development. Epigenetic marks for NPC diagnosis are urgently needed. In order to explore DNA methylation marks, we investigated DNA methylation of candidate genes in EBV-associated nasopharyngeal carcinoma.

**Methods:**

We first employed methyl-capture sequencing and cDNA microarrays to compare the genome-wide methylation profiles of seven NPC tissues and five non-cancer nasopharyngeal epithelium (NNE) tissues. We found 150 hypermethylated CpG islands spanning promoter regions and down-regulated genes. Furthermore, we quantified the methylation rates of seven candidate genes using bisulfite amplicon sequencing for nine NPC and nine NNE tissues.

**Results:**

All seven candidate genes showed significantly higher methylation rates in NPC than in NNE tissues, and the ratios (NPC/NNE) were in descending order as follows: *ITGA4* > *RERG* > *ZNF671* > *SHISA3* > *ZNF549* > *CR2* > *RRAD*. In particular, methylation levels of *ITGA4, RERG,* and *ZNF671* could distinguish NPC patients from NNE subjects.

**Conclusions:**

We identified the DNA methylation rates of previously unidentified NPC candidate genes. The combination of genome-wide and targeted methylation profiling by next-generation sequencers should provide useful information regarding cancer-specific aberrant methylation.

**Electronic supplementary material:**

The online version of this article (doi:10.1186/s12885-017-3482-3) contains supplementary material, which is available to authorized users.

## Background

Molecular fingerprints, including methylation changes, occur in specific human genes following exposure to environmental carcinogens [1]. Epigenetic changes play a crucial role in carcinogenesis [2]. Such epigenetic alterations include hypermethylation of CpG islands in gene promoter regions. DNA hypermethylation serves as a mechanism for inactivation of tumor suppressor genes (TSGs) in human malignancies. Nasopharyngeal carcinoma (NPC) is a rare malignancy in Western countries, but it is endemic and has become a serious health problem in Southeast Asia and southern China [3]. DNA methylation is a common event in Epstein-Barr virus (EBV)–associated NPC, and a number of tumor suppressor genes were found to be silenced or down-regulated in NPC [4–6]. Aberrant DNA methylation, especially in TSG promoters, may be useful as a biomarker [7] for the early diagnosis and prognosis of NPC.

Genome-wide mapping of DNA methylation is essential to identify new disease genes and potential drug targets, as it can reveal many novel regions with epigenetic alterations in disease and provide a rich source of potential biomarkers [8]. Methyl-capture sequencing (Methyl-Cap sequencing) is a robust DNA methylation profiling approach that is based on the capture of methylated DNA using the high-affinity methyl-CpG binding domain of human MBD2 protein and subsequent next-generation sequencing analysis of enriched fragments. Methyl-Cap sequencing is theoretically able to identify methylated genomic regions located anywhere in the genome. However, certain sequential screening methods are required to establish an informative biomarker panel.

A novel method termed bisulfite amplicon sequencing (BAS), which combines the benefits of bisulfite conversion, targeted amplification, and next-generation sequencing, was developed for targeted digital quantitation of DNA methylation [9]. BAS allows for focused, accurate DNA methylation quantitation with high-throughput capabilities in both sample and target numbers. The application of BAS is useful in hypothesis-driven epigenetic studies where regions of interest have been identified [9]. Here we identify novel DNA methylation biomarker candidates for NPC using Methyl-Cap sequencing and BAS.

## Methods

### Clinical samples

Methyl-Cap sequencing was performed on seven tumor biopsies from untreated NPC patients (mean age ± SD, 49.4 ± 6.7 years old; four males, three females) and five non-cancer nasopharyngeal epithelium (NNE) samples from control (non-cancer) patients (47.7 ± 10.4 years old, two males, three females). BAS analysis was conducted using nine NPC samples (45.4 ± 12.1 years old, six males, three females) and nine NNE samples (39.8 ± 13.9 years old, five males, four females). All NPC samples were non-keratinizing carcinoma. The diagnoses were made by experienced pathologists according to the World Health Organization (WHO) classification. All samples were obtained from patients seen at the Department of Otolaryngology Head & Neck Surgery, First Affiliate Hospital of Guangxi Medical University, Nanning, China (with ethical review committee approval notice (2009–07-07) of the First Affiliated Hospital of Guangxi Medical University and ethical approval (no.1116) of Mie University, Japan). All patients provided written informed consent. Biopsy samples were stored in liquid nitrogen prior to DNA or RNA extraction. The tissues of all NPC patients were EBV positive and those of all NNE patients were EBV negative.

### Cell culture

NPC cell line HK1_EBV and immortalized nasopharyngeal epithelial cell line NP460 were the kind gifts of Professor Sai-Wah Tsao (Hong Kong University) [10, 11]. HK1_EBV cells were maintained in RPMI 1640 medium (Gibco, 11,875–093) supplemented with 10% fetal bovine serum (Biowest, S1820), 100 U/ml penicillin, and 100 μg/ml streptomycin (Gibco, 15,070–063). NP460 cells were maintained in a 1:1 ratio of Defined Keratinocyte-SFM (Gibco, 10,744,019) supplemented with growth factors and EpiLife medium supplemented with EpiLife Defined Growth Supplement (Gibco, #S-012-5), 100 U/ml penicillin, and 100 μg/ml streptomycin. Cells were maintained at 37 °C in a 5% CO_2_ incubator.

### Methyl-Cap sequencing

Genomic DNA from frozen tissues and cultured cells was extracted using a QIAamp DNA Mini Kit (Qiagen, 51,304). DNA was sonicated to yield the desired size range (150 bp) using an ultra-sonicator (Covaris, Woburn, MA). After sonication, methylated DNA was selected from 12.5-μg DNA fragments using a MethylMiner Methylated DNA Enrichment Kit (Invitrogen, ME10025). We collected the final two fractions of highly methylated DNA, which corresponded to gradient elution buffer concentrations of 0.6 M and 2 M NaCl. The recovered DNA in the 2 M NaCl elution buffer was purified with a PureLink PCR Purification Kit (Invitrogen, K3100–02). Library construction, emulsion PCR, and sequencing were performed by Mie University Life Science Research Center using a SOLiD System (Applied Biosystems, Foster City, CA) with mapping to the human reference genome (hg 19). Partek Genomics Suite (Partek Incorporated, Saint Louis, MO) was used to map BAM files to the human CpG islands for further statistical analyses. We checked the methylation status using the Integrative Genomics Viewer (IGV) (ver1.4.05), as shown in Additional file 1: Figure S1.

### Detection of gene expression using cDNA microarray analysis

Fifty nanograms of RNA from seven NPC biopsies and five NNE samples (Methyl-Cap sequencing samples) were subjected to Agilent SurePrint G3 Human GE microarray analysis (8 × 60 K, 1 color, Agilent Technologies, Santa Clara, CA) for gene expression evaluation (Hokkaido System Science).

### Sodium bisulfite modification and bisulfite sequencing PCR

Genomic DNA (1 μg) from each sample was treated with sodium bisulfite using an EpiTect Bisulfite Kit (Qiagen, 59,104) and QIACube (Qiagen). Sodium bisulfite–modified DNA was subjected to PCR with bisulfite sequencing PCR primers, which were designed to amplify nucleotides in CpG islands around the transcription start sites of target genes. The primer sequences and cycling conditions for BAS are listed in Table 1. PCR products were purified using the PureLink PCR Purification Kit (Invitrogen). The purified products from individual biological samples were pooled in equimolar amounts (0.5 pmol) of 10 genes from each subject (approximately 1 μg/sample), including the seven target genes in this study.

**Table 1:** Bisulfite sequencing PCR primers and PCR conditions for BAS samples

Gene	Sequences (5′ to 3′)	Product size (bp)	Annealing (°C)	Annealing Time (s)	Position from TSS	UCSC gene ID
*CR2*	F: GGGTGAGTTTGAGTTAAAGAGTGG R: AAAAAACCAATAAAAACAATCAAAACCAAA	514	58	50	−149 – +365	uc001hfv.3
*ITGA4*	F: TGTAATTTTGGGGTAGTGGT R: CCCTCCTACCTCCTTAAAAAAAAAAAA	362	58	45	+711 − +1072	uc002unu.3
*RERG*	F: GGAGTTTGGAGGTTTGGAAAT R: CAAAAACAAATACCAATAACCC	278	58	45	−145 – +133	uc001rct.3
*RRAD*	F: TTGGTGGGGGTGGATAGATA R-CCTCCCCCAACCCCCAAAT	331	61	45	−102 – +228	uc002eqo.2
*SHISA3*	F: GGTTGAGAGTTAAGTTTTGGGGG R: CCTCCCCACTCCTCAAAAAAA	446	58	45	−570 – −125	uc003gwp.3
*ZNF549*	F: TTTTAGTTTGATGGGTTTTTTTTTTTGTT R: AAACCTCAAAACCCAAATAAAAATC	502	56	45	−177 – +325	uc002qpb.2
*ZNF671*	F: ATTTTGTTTTTGTTAGGTTGTTTTTGG R: CTATCCTAAAACACAAAAACTACAAACACT	311	57	45	+13 − +323	uc002qpz.4

PCR cycles: 40

*CR2*: complement C3d receptor 2, *ITGA4*: integrin subunit alpha 4, *RERG*: RAS-like estrogen regulated growth inhibitor, *RRAD*: Ras-related glycolysis inhibitor and calcium channel regulator, *SHISA3*: shisa family member 3, *ZNF549*: zinc finger protein 549, *ZNF671*: zinc finger protein 671

### Bisulfite amplicon sequencing (BAS)

Pooled PCR products were sheared using the Ion Shear Plus Enzyme Mix to yield appropriate insert sizes, and transformed with the Ion Xpress Plus Library kit for AB Library Builder System (Life Technologies, Carlsbad, CA) into barcoded libraries with sizes set at 200 bp. Emulsion PCR was performed using the Ion PGM Hi-Q OT2 Kit, and sequenced using the Ion PGM Hi-Q Sequencing Kit on Ion PGM (400-bp read length) with a 318 Chip v2 BC (Life Technologies). Sequencing data were analyzed using the Bismark Bisulfite Mapper [12] with plug-in software (Life Technologies). The percent methylation in each CpG was calculated by (number of reads with methylated C/total reads) × 100. The methylation data can be viewed in the IGV using the BiSeq package in R/Bioconductor.

### Bisulfite genomic sequencing (BGS)

To compare the BAS and BGS methods, sodium bisulfite–modified DNA was subjected to PCR with bisulfite sequencing primers for *ITGA4* and *ZNF549*, shown in Table 1, with an annealing time of 30 s. Subcloning and sequencing for BGS were performed as described previously [4].

## Results

### Selection of candidate genes with hypermethylated promoter CpG islands and reduced expression in NPC tissues

First, we targeted promoter CpG islands with overlapping regions from 1000 bp upstream to 200 bp downstream of each gene’s transcription start site (about 23,000 genes). Next, we selected 150 candidate genes with hypermethylated promoter CpG islands (more than 3-fold based on Methyl-Cap sequencing data, *P* < 0.05) and down-regulated genes (relative quantity less than 0.5 based on cDNA microarray data, *P* < 0.05) in NPC compared to NNE tissues (Additional file 2: Table S1). We performed a literature search on DNA methylation in these genes, and finally chose seven genes (Table 1) for further study.

### Comparison of methylation rates between BAS and BGS

**Fig. 1:**
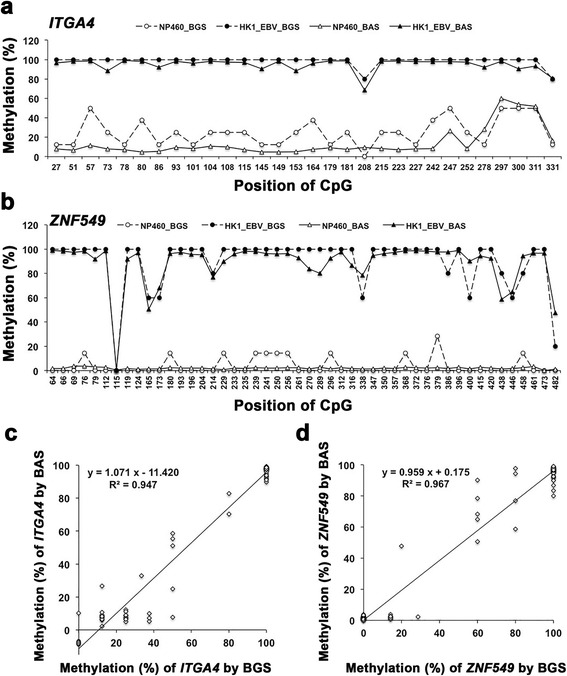
Comparison of CpG methylation rates between BGS and BAS. The methylation rate of each CpG in promoter regions of *ITGA4* (**a**) and *ZNF549* (**b**) was detected by BGS (circles, dotted line) and BAS (triangles, solid line) in HK1_EBV cells (closed marker) and NP460 cells (open marker). Graphs show the correlation in methylation rates between BGS and BAS for *ITGA4* (**c**) and *ZNF549* (**d**) with Pearson’s correlation coefficients

Using BAS, the average read depth per CpG (mean ± SD, 1274.6 ± 295.0; range 961.7–1707.1) for seven genes in 20 samples was sufficient to estimate the methylation rate. The bisulfite sequencing PCR amplicons of *ITGA4* and *ZNF549* from non-cancer cell line NP460 and NPC cell line HK1_EBV were also subjected to BGS, and at least five clones were successfully evaluated for all CpG methylation statuses (Additional file 1: Figure S2). From both sequencing results, the methylation rate in every CpG was calculated as shown in Fig. 1. Compared to NP460 cells, HK1_EBV cells were more highly methylated in the promoter CpGs of *ITGA4* (Fig. 1a) and *ZNF549* (Fig. 1b). The scatter plots show good correlation in methylation rates between BGS and BAS (*ITGA4*, *R* = 0.973, *P* = 0.000, Fig. 1c; *ZNF549*, *R* = 0.983, *P* = 0.000, Fig. 1d).

### Methylation quantification of promoter CpGs by BAS

**Fig. 2:**
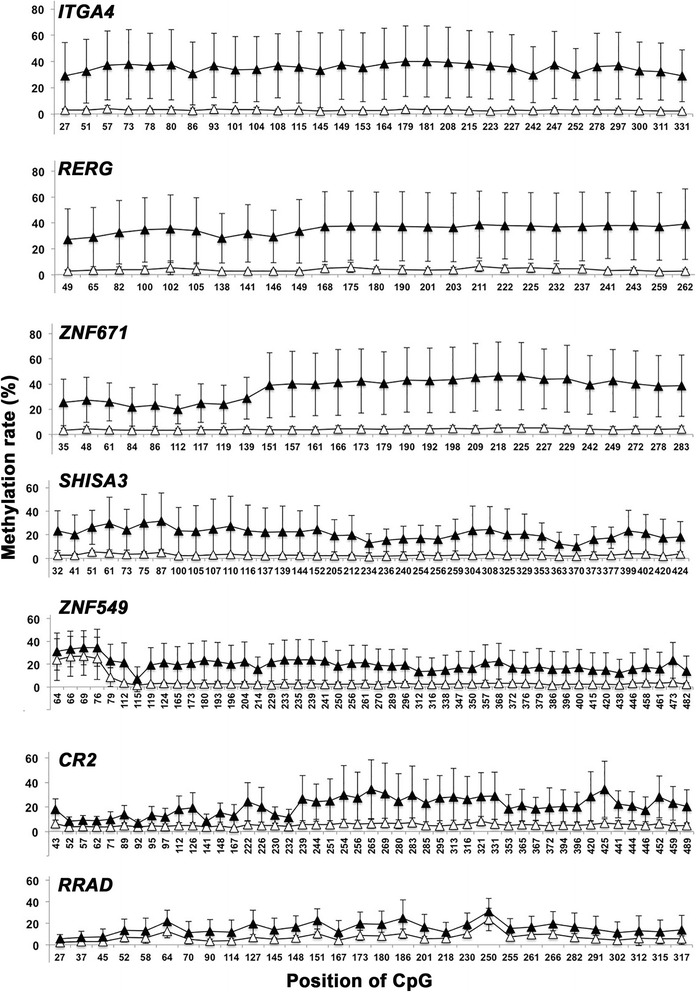
CpG methylation rates in NPC and NNE tissues. Graphs show mean and SD (%) in every CpG from NPC (*n* = 9, closed triangles) and NNE (*n* = 9, open triangles)

Table 2 shows the DNA methylation rates derived by BAS analysis for NNE and NPC patients. All seven genes exhibited significant differences in DNA methylation rates between NNE and NPC patients. Among the seven genes, the ratios of the methylation rates in the two groups (NPC/NNE) were, in descending order, *ITGA4* > *RERG* > *ZNF671* > *SHISA3* > *ZNF549* > *CR2* > *RRAD*. Fig. 2 shows the methylation rate of every CpG (average and SD, %) for the NPC and NNE subjects. High methylation rates were observed in NPC patients (closed triangles) compared with NNE patients (open triangles), especially for *ITGA4*, *RERG*, and *ZNF671*.

**Table 2:** DNA methylation rate by BAS analysis

	No. CpG	NNE (*n* = 9) Mean ± SD (%)	NPC (*n* = 9) Mean ± SD (%)	*P*-value by t-test	Ratio of NPC/NNE (rank^a^)
*CR2*	47	5.3 ± 2.6	20.7 ± 11.7	0.004	3.9 (6)
*ITGA4*	30	3.0 ± 1.1	35.3 ± 24.6	0.004	11.8 (1)
*RERG*	25	3.9 ± 2.2	35.2 ± 25.0	0.006	9.1 (2)
*RRAD*	30	7.1 ± 2.5	15.3 ± ﻿9.9	0.038	2.2 (7)
*SHISA3*	37	2.9 ± 0.7	21.1 ± 15.3	0.007	7.3 (4)
*ZNF549*	48	4.7 ± 2.1	19.7 ± 13.5	0.010	4.2 (5)
*ZNF671*	28	4.1 ± 2.0	36.4 ± 20.7	0.002	8.9 (3)

^a^: rank in descending order

### Visualization of methylation status at a glance

**Fig. 3:**
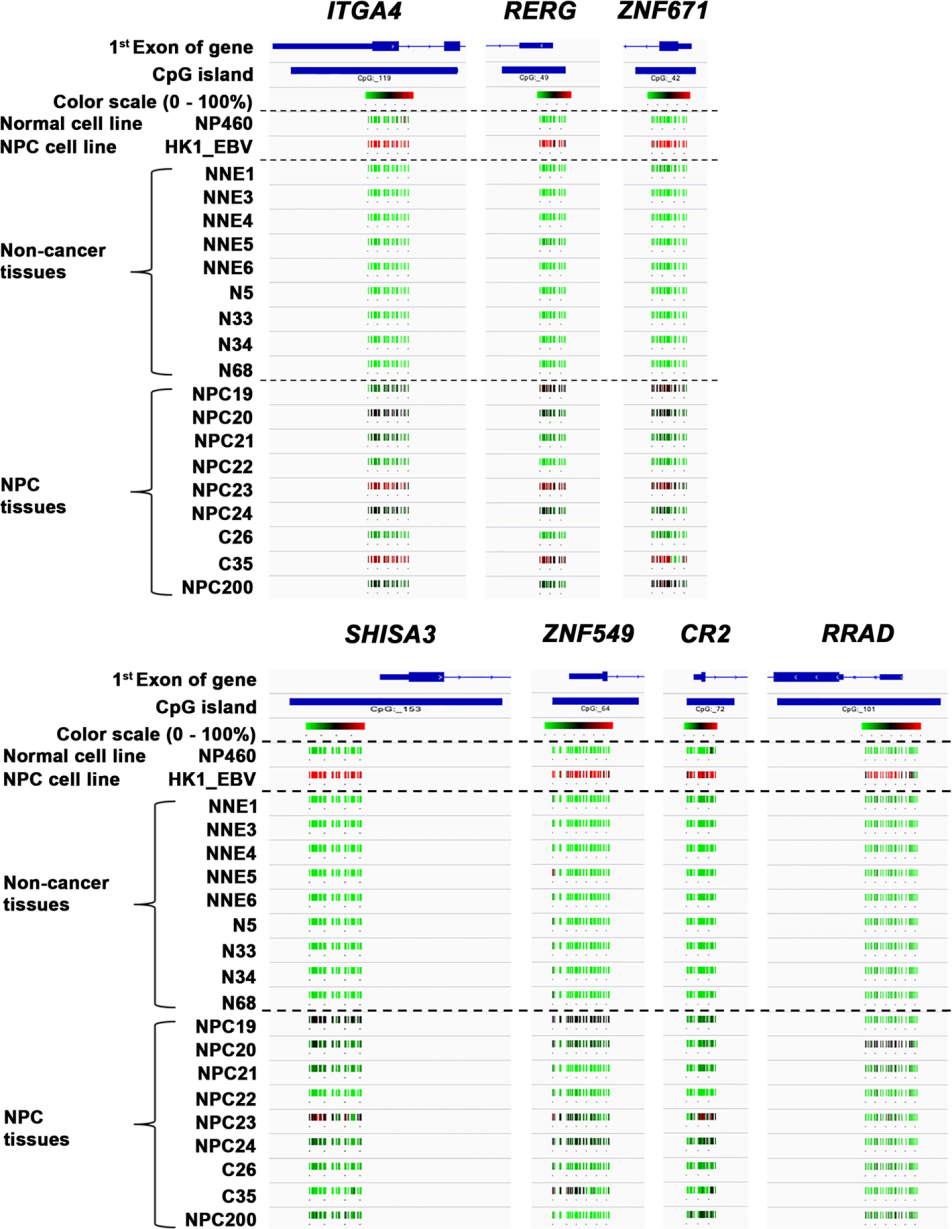
Methylation rates of NPC and NNE cell lines and tissues depicted with a color scale. Integrative Genomics Viewer data shows the first exons of genes and CpG islands, and the color scale represents the PCR-amplified region. Color scale varies from green to red, corresponding to 0% – 100% methylation rates

BAS data were analyzed using the Bismark Bisulfite Mapper with plug-in software and the BiSeq package in R/Bioconductor, and then the resultant BED files were visualized by IGV. Fig. 3 shows the methylation status of each subject using a color scale based on methylation rate (green 0% — black 50% — red 100%), making it possible to detect differences in methylation status at a glance.

## Discussion

Epigenetic changes such as DNA methylation are recognized as an important mechanism in cancer initiation and progression [13]. Inactivation of TSGs occurs as a consequence of promoter hypermethylation with gene silencing in many cancer types [14]. In NPC, a vast number of TSGs have been found to be inactivated by promoter hypermethylation [15]. Interestingly, EBV infection induces increased genome-wide gene methylation, resulting in the formation of a unique epigenotype with high CpG methylation in tumor cells [16]. Given its important functions in cancer initiation and progression, DNA methylation is being explored as a biomarker for cancer, including NPC.

Many methods are available to examine DNA methylation at single-base resolution. These are broadly classified into two categories, depending on whether they are based on microarrays or next-generation sequencing. Microarray-based technologies use a fixed number of probes with the limitation of low genome coverage and the advantage of low cost [17]. Whole-genome bisulfite sequencing can overcome this limitation but elevates the costs tremendously [17]. It is only practical to conduct whole-genome bisulfite sequencing on a limited number of samples, and coverage is usually in the range of 5–15 reads per CpG, limiting the statistical significance of results [18]. Methyl-Cap sequencing is an attractive intermediate solution to increase the methylome coverage in large sample sets [17]. We utilized Methyl-Cap sequencing and cDNA microarray analysis to explore TSG candidates with highly methylated promoter CpG islands and gene down-regulation, resulting in 150 possibilities. Of these 150 candidate genes, several had already been reported to be epigenetic silencing of tumor suppressor genes in NPC, such as *ZFP82* [19], *ADAMTS8* [20], *INPP4B* [21], and *ATOH8* [22]. Several conventional NPC tumor suppressor genes [23], such as *RASSF1* and *CDKN2A* (*p16*), were methylated at promoter regions in NPC patients from our Methyl-Cap sequencing data, but their expression levels were not significantly down-regulated in NPC by cDNA microarray analysis. Gene *ZMYND10* (*BLU*) was significantly down-regulated in NPC, but there was no significant difference in Methyl-Cap sequencing between NNE and NPC (data not shown). Since we combined Methyl-Cap sequencing (more than 3-fold) and cDNA microarray (less than 0.5-fold) data, these TSGs were not included in our candidate gene list (Additional file 2: Table S1). Our literature review resulted in the selection of seven target genes. These genes were previously reported to exhibit DNA methylation (*CR2* [24], *ITGA4* [25], *RERG* [26], *RRAD* [4], *SHISA3* [27], *ZNF549,* and *ZNF671* [28]). Schwab and Illges found that premature B lymphocytes contained a methylated CpG island and did not express *CR2* (*CD21*) [24]. Interestingly, viral capsid protein mediated EBV binding on CR2 [29]. Gerecke et al. showed that methylation markers in the promoters of *ITGA4*, *TFPI2*, and *VIMENTIN* seemed to be suitable risk markers for inflammation-associated colon cancer [25], and Chang et al. demonstrated *ITGA4*, *SFRP2*, and *p16* promoter methylation in stool samples from patients with colorectal adenomas and carcinoma [30]. *RERG* was reported to be a tumor suppressor gene in colorectal cancer [31] and breast cancer [26]. Our previous study demonstrated that *RRAD* was frequently methylated in EBV-associated NPC, and it functioned as a tumor suppressor by inhibiting cell proliferation, colony formation, and migration in *RRAD*-overexpressing NPC cells [4]. *SHISA3* was a novel tumor suppressor identified in lung cancer [32], and was found to be epigenetically inactivated in a substantial fraction of patients with colorectal cancer [27]. Yeh et al. demonstrated that *ZNF671*, an epigenetically silenced novel tumor suppressor, was a potential non-invasive biomarker for predicting urothelial carcinoma relapse [33]. Lleras et al. reported the epigenetic silencing of Kruppel-type zinc finger protein genes, including *ZNF549* and *ZNF671*, on chromosome 19q13 in oropharyngeal cancer [28]. Our results demonstrated the utility of BAS in validating findings from genome-wide methylation analysis, by showing that all seven candidates had significantly higher average CpG methylation rates in NPC than NNE.

BAS is an efficient, cost-effective, and robust high-throughput technique for assessing DNA methylation at targeted loci of interest [18]. In our experiment, BAS coverage attained an average of over 1000 reads per CpG. BGS is another method of targeted bisulfite sequencing that includes subcloning and clone selection steps, which limits the total numbers of sequenced clones and sample sets [34]. Therefore, BGS coverage is usually in the range of around 10 clones per CpG. Due to the significantly increased throughput of next-generation sequencing, a large number of differentially methylated genes can now be identified in a single experiment, and the traditional methods of experimental validation, such as methylation-specific PCR and BGS, are no longer sufficient to keep up with increasing demand. As our results show, BAS can quantitatively and accurately measure CpG methylation levels in genomic regions of interest in a high-throughput manner, and this approach may replace traditional validation methods in the future.

## Conclusions

Here we show seven candidate epigenetic marks for NPC (methylation ratios: *ITGA4* > *RERG* > *ZNF671* > *SHISA3* > *ZNF549* > *CR2* > *RRAD*). In conclusion, the combination of genome-wide and targeted methylation profiling by next-generation sequencers provides useful information regarding cancer-specific aberrant methylation in NPC.

## Additional files

Additional file 1: Figure S1. DNA methylation data visualized with IGV. This figure presents the result of methylation analysis by Methyl-Cap sequencing at promoter regions of SHISA3 in NPC and NNE sample, respectively. **Figure S2.** Methylation status of promoter regions in cell lines. Bisulfite genomic sequencing of 30 and 48 CpG sites within the promoter regions of (A) ITGA4 and (B) ZNF549, respectively, in an immortalized epithelial cell line (NP460) and an NPC cell line (HK1_EBV). At least five clones were randomly selected and sequenced for each sample. Each row represents an individual promoter allele. Open circles indicate unmethylated cytosines, and closed circles indicate methylated cytosines. (ZIP 584 kb)

Additional file 2: Table S1. Summary of candidate genes from Methyl-Cap sequencing and cDNA microarray data. (XLSX 24 kb)

## Abbreviations

*ADAMTS8*: ADAM metallopeptidase with thrombospondin type 1 motif 8
*ATOH8*: Atonal bHLH transcription factor 8
BAS: Bisulfite amplicon sequencing
BGS: Bisulfite genomic sequencing
*CDKN2A* (*p16*): Cyclin dependent kinase inhibitor 2A
*CR2*: Complement C3d receptor 2
IGV: Integrative Genomics Viewer
*INPP4B*: Inositol polyphosphate-4-phosphatase type II B
*ITGA4*: Integrin subunit alpha 4
Methyl-Cap sequencing: Methyl-capture sequencing
NNE: Non-cancer nasopharyngeal epithelium
NPC: Nasopharyngeal carcinoma
*RASSF1*: Ras association domain family member 1
*RERG*: Ras-like estrogen-regulated growth inhibitor
*RRAD*: Ras-related glycolysis inhibitor and calcium channel regulator
*SHISA3*: Shisa family member 3
TSG: Tumor suppressor gene
*ZFP82*: ZFP82 zinc finger protein
*ZMYND10* (*BLU*): Zinc finger MYND-type containing 10
*ZNF549*: Zinc finger protein 549
*ZNF671*: Zinc finger protein 671
