# Supplementary figures and images for: Quantitation of DNA methylation in Epstein-Barr virus–associated nasopharyngeal carcinoma by bisulfite amplicon sequencing
Source: BMC Cancer. 2017 Jul 17;17:489. doi: 10.1186/s12885-017-3482-3 (PMC5514474; doi:10.1186/s12885-017-3482-3)

## Slide 1
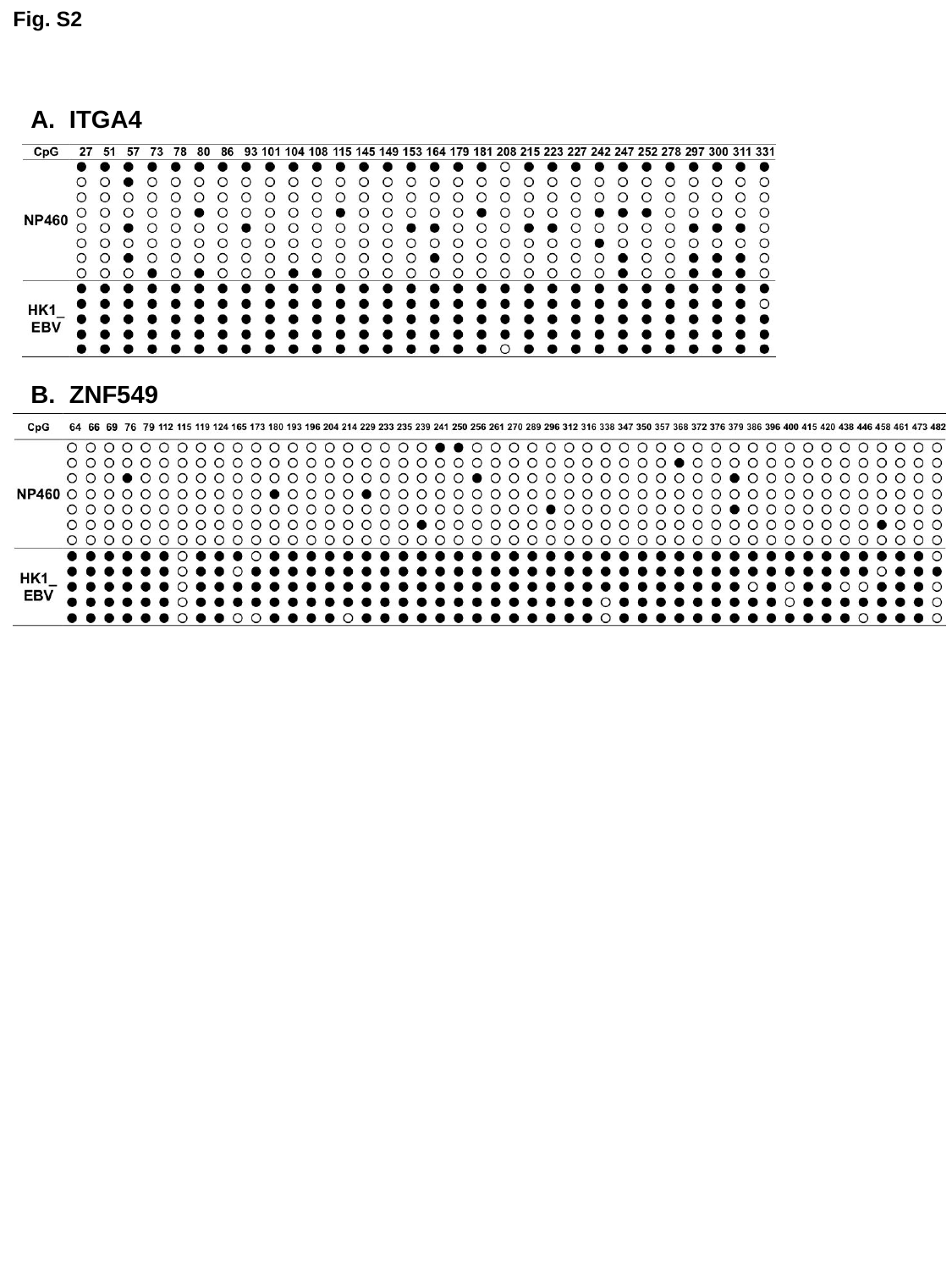

Fig. S2
A. ITGA4
B. ZNF549

Supplement: Supplementary file 1 — DNA methylation data visualized with IGV. This figure presents the result of methylation analysis by Methyl-Cap sequencing at promoter regions of SHISA3 in NPC and NNE sample, respectively. Figure S2. Methylation status of promoter regions in cell lines. Bisulfite genomic sequencing of 30 and 48 CpG sites within the promoter regions of (A) ITGA4 and (B) ZNF549, respectively, in an immortalized epithelial cell line (NP460) and an NPC cell line (HK1_EBV). At least five clones were randomly selected and sequenced for each sample. Each row represents an individual promoter allele. Open circles indicate unmethylated cytosines, and closed circles indicate methylated cytosines. (ZIP 584 kb) [file 12885_2017_3482_MOESM1_ESM.zip › Fig.S2 BMC Cancer fR2.pptx]

## Slide 1
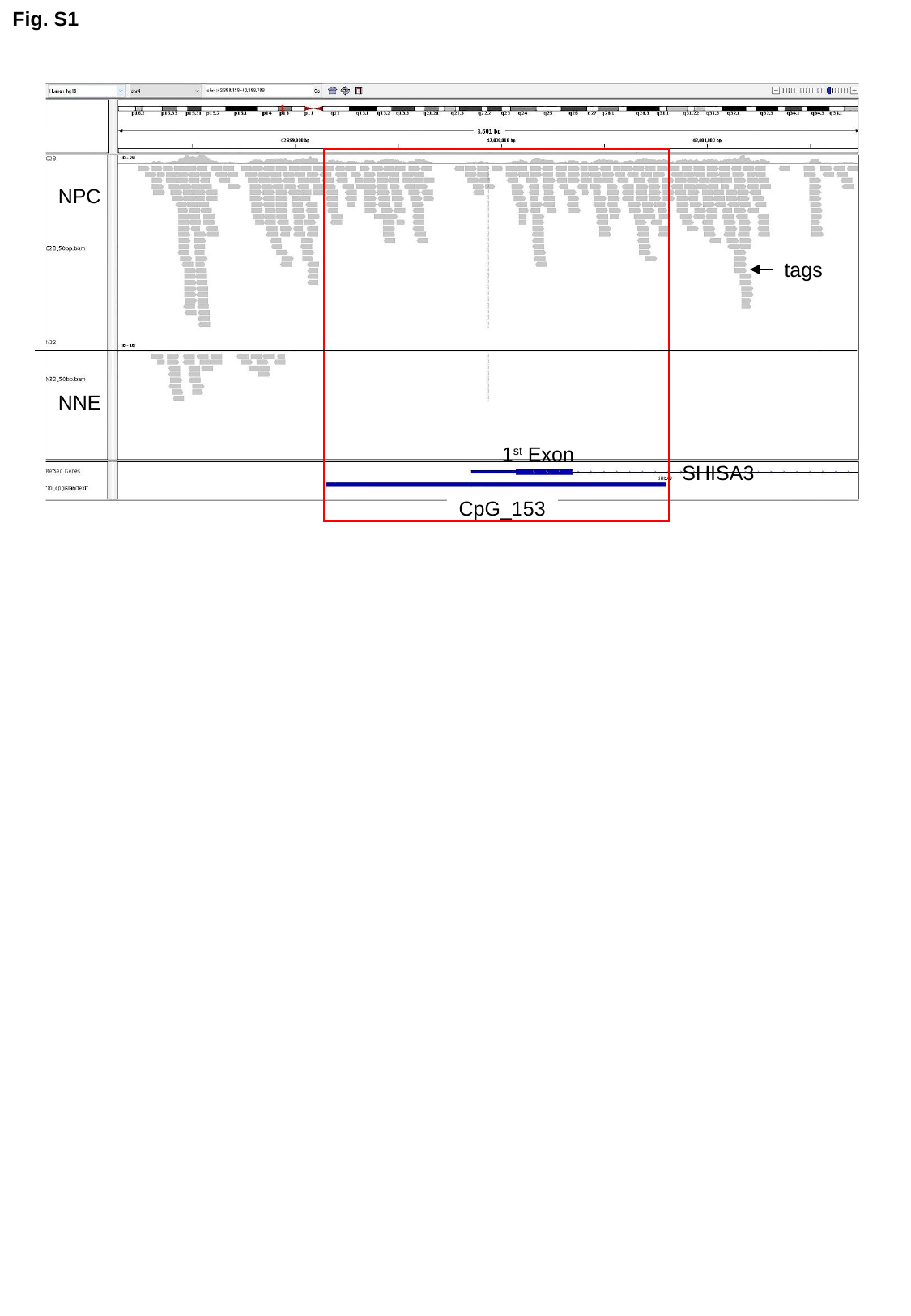

Fig. S1
NPC
 tags
NNE
1st Exon
SHISA3
CpG_153

Supplement: Supplementary file 1 — DNA methylation data visualized with IGV. This figure presents the result of methylation analysis by Methyl-Cap sequencing at promoter regions of SHISA3 in NPC and NNE sample, respectively. Figure S2. Methylation status of promoter regions in cell lines. Bisulfite genomic sequencing of 30 and 48 CpG sites within the promoter regions of (A) ITGA4 and (B) ZNF549, respectively, in an immortalized epithelial cell line (NP460) and an NPC cell line (HK1_EBV). At least five clones were randomly selected and sequenced for each sample. Each row represents an individual promoter allele. Open circles indicate unmethylated cytosines, and closed circles indicate methylated cytosines. (ZIP 584 kb) [file 12885_2017_3482_MOESM1_ESM.zip › Fig S1 BMC Cancer fR2.pptx]
